# Planning the oral health workforce: Time for innovation

**DOI:** 10.1111/cdoe.12604

**Published:** 2020-12-15

**Authors:** Stephen Birch, Susan Ahern, Paul Brocklehurst, Usuf Chikte, Jennifer Gallagher, Stefan Listl, Ratilal Lalloo, Lucy O'Malley, Janet Rigby, Martin Tickle, Gail Tomblin Murphy, Noel Woods

**Affiliations:** ^1^ Centre for the Business and Economics of Health University of Queensland Brisbane Queensland Australia; ^2^ Centre for Health Economics University of Manchester Manchester UK; ^3^ Oral Health Services Research Centre Cork University Dental School & Hospital University College Cork Cork Ireland; ^4^ School of Health Sciences Bangor University Bangor UK; ^5^ Department of Global Health Stellenbosch University Cape Town South Africa; ^6^ Faculty of Dentistry Oral & Craniofacial Sciences King's College London London UK; ^7^ Department of Dentistry ‐ Quality and Safety of Oral Healthcare Radboud University Nijmegen The Netherlands; ^8^ School of Dentistry University of Queensland Brisbane Queensland Australia; ^9^ School of Dentistry University of Manchester Manchester UK; ^10^ WHO/PAHO Collaborating Centre on Health Workforce Planning & Research Dalhousie University Halifax NS Canada; ^11^ Centre for Policy Studies Cork University Business School University College Cork Cork Ireland

**Keywords:** health, health service needs and demands, policy, population

## Abstract

The levels and types of oral health problems occurring in populations change over time, while advances in technology change the way oral health problems are addressed and the ways care is delivered. These rapid changes have major implications for the size and mix of the oral health workforce, yet the methods used to plan the oral health workforce have remained rigid and isolated from planning of oral healthcare services and healthcare expenditures. In this paper, we argue that the innovation culture that has driven major developments in content and delivery of oral health care must also be applied to planning the oral health workforce if we are to develop ‘fit for purpose’ healthcare systems that meet the needs of populations in the 21st century. An innovative framework for workforce planning is presented focussed on responding to changes in population needs, service developments for meeting those needs and optimal models of care delivery.

## 1 INTRODUCTION

The levels and types of oral health problems occurring in populations have changed and will continue to change over time with, for example, large declines in the rates of prevalence of dental disease experienced in high‐income countries over the last 30 years.^1^ Similarly, innovations and advances in technology have and will continue to change the way oral health problems are addressed (eg, service developments such as fluoride varnishes),^2^ and the ways care is delivered (new models of provision involving changes in skill mix^3^, ^4^, ^5^, ^6^) and funded (changes in public coverage and the public/private mix of service delivery^7^, ^8^). These often rapid and continuing changes, together with further calls for changes to the oral health sector,^9^, ^10^ have major implications for the size and mix of the oral health workforce. Yet, little if any attention is paid to reflecting these changes in policy objectives for planning and managing the oral health workforce. In this paper, we show that market forces are unlikely to respond to these changes in ways that create the capacity to meet needs for care in the population and ensure those resources are used efficiently. However, in many jurisdictions little attention is given to planning the future oral health workforce, while in others the methods used to plan the workforce have remained rigid and isolated from planning of oral healthcare services and planning expenditures for those services.^11^ To continue planning the oral health workforce as we currently do will not provide a sustainable solution to meeting the needs of the population. In this paper, we argue that the innovation culture that has driven major developments in content and delivery of oral health care must also be applied to planning the oral health workforce. If we are to realize the gains from such innovations in healthcare delivery and have ‘fit for purpose’ healthcare systems that meet the needs of populations in the 21st century, (a) workforce planning must be integrated with service delivery planning and the allocation of resources to oral health care, and (b) planning frameworks must be dynamic, responding to changes in population health and advances in oral health service delivery.

## 2 WHY PLANNING THE ORAL HEALTH WORKFORCE IS REQUIRED?

In many instances, labour markets are left to determine the number and type of workers through ‘market forces’ in the form of variable wage rates, ensuring that surpluses or shortages are easily and quickly removed. The demand for workers is derived from the goods and services that consumers demand and workers produce. In the case of healthcare markets however, some of the underlying assumptions of this ‘free‐market’ model are not valid and government intervention is required, if the number and type of human resources delivering services are to be sufficient to meet the objectives of the healthcare system and used in their most productive ways. For example, the assumption that the services providers produce will be consumed by those with greatest need does not hold. Hence, the overall health gains from the services produced are not maximized. This is referred to as the ‘inverse care law’.^12^ In addition, the assumption that the demand for the services produced by providers is determined exclusively by consumers/patients and hence independent of the influence of providers also does not hold.^13^, ^14^, ^15^, ^16^ Consumers/patients are concerned with their levels of oral health and risks to health, and their demand is for changes in those levels. Because knowledge about oral health and health care is complex, and the risk of harm to the public is significant, consumers/patients seek the advice of health professionals to determine what services are required to achieve their desired changes in health status (eg, eliminate pain) or risks to health (reduce the chance of future pain). Given this information asymmetry between individuals and providers, the demand for dentists will be *derived* from the demand for the services that dentists prescribe and provide for individuals' demanding changes in health and health risks. As a result, the demand for services is determined at least in part by suppliers' interests and may not represent only the consumers' best interests, or the distribution of services in the population required to maximize health gain.^16^, ^17^

Because consumers/patients are not in a position to know what services they need, and may not be in a position to purchase those services, resulting from their limited levels of income and wealth, we face ‘market failure’ for oral health care. The result is inefficiency in the levels of services produced and the distribution of those services in the population. This is reflected in the capacity to provide care, with market forces producing the number of providers to meet demands, irrespective of the levels and distribution of needs. Market failure refers to the failure of the ‘system’ to deliver care where it is most effective, not the failure of individual providers to deliver high‐quality care to presenting patients.^18^

Governments intervene in healthcare markets in response to these market failures with the intention of promoting more efficient use of healthcare resources. In the absence of market forces delivering the ‘right’ amount of care to the ‘right’ populations, governments take on the role of producing and distributing the capacity to care (ie, providers and healthcare facilities) in accordance with the needs for care in the population. In this way, the capacity to care reflects the differences in needs for care across populations and responds to changes in those needs over time. Government programmes in the market for oral health care are often limited to something less than ‘universal coverage’ and focus on specific population groups (eg, children, the elderly or low‐income populations). However, the importance of careful strategic planning of the workforce remains if government funds are to be used in ways that support those programmes making greatest impact on reducing the needs of those populations covered.

The development of a ‘needs‐based, needs responsive’ system is not a natural consequence of government intervention in the market. Instead, governments must adopt policies and strategies that provide the needs‐based direction that market forces fail to provide. Otherwise, market failure is simply replaced by government failure—healthcare providers continue to deliver services and serve patients in ways that meet expressed demands, but fail to achieve maximum impact on population needs for care.

Concern with access to services among those in greatest need has generally been addressed through policies on removing, or reducing, the cost of care to patients by subsidizing prices patients pay at the point of service delivery.^19^ While this removes the price barrier to patients when care is needed, it has no effect on ensuring the capacity to care will be sufficient to meet those needs, or will be redirected towards areas of greatest needs. Hence, policies such as universal health coverage, aimed at enabling those with needs to demand care, require policies that provide and direct capacities to care to meet those needs. Being able to demand care when care is needed will have limited success if the capacity to care is not there to satisfy those demands. Policies aimed at promoting the demand for care in accordance with needs therefore require complementary policies, aimed at planning capacities to care that reflect the distribution of needs in the population, and changes in those needs over time.^20^ In this way, needs‐based access to care requires needs‐based planning of capacities to care and in particular, needs‐based plans for the workforce to deliver that care.

## 3 TRADITIONAL APPROACHES TO HEALTH WORKFORCE PLANNING

Workforce planning involves comparing the expected future availability (or ‘supply’) of providers with the expected future need (or ‘demand’) for providers and developing policies to address any expected gaps (surpluses or shortfalls) between availability and need. Traditionally, health workforce planning has been based on applying forecasted changes in demography (the size and age distribution of the population) to the current levels of supply, measured by the ratio of number of providers to the size of the population (provider‐population ratio), or current levels of utilization, measured by the ratio of services used to the size of the population (utilization‐population ratio).^21^ Under these approaches, a population that is expected to grow by 5% over the next 2 years will require a 5% increase in the number of providers or quantity of services. Health policymakers or policy planners may respond to concerns with waiting times by adopting a higher provider‐population or utilization‐population ratio, or simply adopt a different ratio value to be in line with ‘international standards’ promoted by agencies such as the World Health Organization, through some target provider‐population ratio. These approaches are demographically driven by ‘projecting the present position’ onto the future population, with no attention given to the characteristics of the population (eg, levels of sickness), the providers (eg, levels of productivity or the quantity of services delivered per provider) or the system (care pathways used).^22^ Essentially, the approach addresses the question *of ‘how many providers will be required in the future to serve the expected future population in the same way as the current population are served?* The approach is simple, but ‘fixed in time’, providing no capacity to consider (a) changes in the needs of populations over time associated with epidemiological transitions, (b) changes in the range and mix of services used to address those needs, or (c) changes in the range and mix of resources (eg, staff, equipment) used to deliver services associated with changes in service mix. Instead, this model of workforce planning is based on implicit assumptions that needs, services and models of care are constant between populations and over time. It generates estimates of the number of providers that would be required for a healthcare system to do precisely that. Hence, the model effectively plans for maintaining or perpetuating any inefficiencies in the current system, in terms of who receives care, what care is received and how care is produced, including levels of unmet need and overutilization.

## 4 FROM POPULATION NUMBER TO POPULATION NEEDS: INNOVATION IN WORKFORCE PLANNING

There is nothing ‘magical’ about current levels of supply or utilization per capita, so there is no reason to adopt these rates for planning the future system or treating these rates as constant moving forward. On the contrary, provider and service ratios are determined by other factors, each of which is variable and can be managed through policy.

The utilization‐population ratio is determined by the level of need per unit population (or epidemiological data on oral health levels and distribution) and the level of services delivered for each level of need (care pathways). The provider‐population ratio is determined by these same two elements together with the number of providers required to deliver a given quantity of services (the inverse of the number of services per provider or productivity). In each case, these determinants represent variables that differ between populations and over time, and can be influenced by policymakers in order to manage the required number of providers in different populations and over time. Changes in epidemiology indicate the level and mix of services required to meet needs will change and that must be reflected in the number and types of provider we plan to use.^23^ So, for example, effective programmes for reductions in childhood caries will reduce the number of providers required to provide care per 100 children, and similarly, a cohort of 100 providers can be expected to manage a greater number of children because of the reduction of prevalence in childhood caries. New evidence on the treatment of caries (eg, changing the criteria for when to restore a tooth) would change the services required to treat a population of 100 children at risk of caries and hence the number of providers required to meet needs in this population. Finally, new technology that increases the throughput of patients, by improving diagnostic methods, would reduce the number of providers required to produce a given quantity of services. For each element, consumer expectations will be important, in terms of understanding need from a patient perspective (eg, patient expectations for oral health, types of intervention and models of delivery) if services are to be demanded and adherence achieved.

These three elements, epidemiology, care pathways and productivity, are independent elements of provider requirements. Changes occurring in any one of the elements have no implications for the other elements. Moreover, they are not simply the result of external factors that the system must accommodate, but are factors that policymakers can influence. Hence, they provide tools for managing the health workforce. Introducing preventive programmes can be used to change the needs of the population; introducing new technology can reduce the quantity of care required to meet needs; and using different skill mixes can reduce the number of providers required to deliver a given quantity of care.

While these examples all reflect positive developments in oral health care (improved epidemiology, better care processes and improved productivity), the model also responds to negative developments should they occur. For example, environmental conditions giving rise to increased prevalence of oral disease would increase the quantity of care required and hence the number of providers to serve the population. The purpose of the model is to provide a framework that is responsive to changes over time, whatever those changes might involve.

The model remains responsive to changes in the size of the population over time, so that any changes in workforce requirements per 100 population, as discussed above, are applied to expected demographic changes in the population, in terms of the number and age distribution of the population in the future. Unlike the elements of needs, care pathways and productivity, which can all be influenced by policy, demography largely lies outside the influence of health policy. However, governments may change policies on public coverage (eg, expanding or restricting coverage within the population) which would affect the requirements for services and workforce through changing the size of the population whose needs are to be addressed by the system. In the next section, we present a ‘fit for purpose’ health workforce planning model that combines changes in demography with changes in epidemiology, care pathways and productivity.

## 5 A ‘FIT FOR PURPOSE’ HEALTH WORKFORCE PLANNING MODEL

The traditional approach to health workforce planning can be summarized as the required number of providers in the future, N_t+1_ being given by,

(1) $$N_{t+1}={\left(N/P\right)}_{t}\times P_{t+1}.$$

where (N/P)_t_: is the current (or target) provider‐population ratio.P_t+1_: is the expected future population.

As argued above, there is nothing ‘optimal’ about the current (or target) ratio (N/P)_t_ and hence no rationale for keeping this constant as the ratio to provide the right services in the right way for the right population. The ratio consistent with achieving that goal would be determined by the separate elements of epidemiology, care pathways and productivity. In other words, (N/P)_t_ can be disaggregated into three separate and independent elements as follows:

(2) $$\left(N/P\right)=\left(N/Q\right)\times \left(Q/H\right)\times \left(H/P\right).$$

where Q: indicates the services we plan to provide in the future.H: indicates the levels of health in the population.

Substituting this expression into Equation 1 gives:

(3) $$N_{t+1}={\left(N/Q\right)}_{t+1}\times {\left(Q/H\right)}_{t+1}\times {\left(H/P\right)}_{t+1}\times P_{t+1}.$$

Under this model the future requirements for providers are given by four variables (or the answers to four policy questions), each of which can be influenced by policy:

P: Population covered—*Who are we caring for?*

If eligibility for coverage changes so do provider requirements.

H/P: Epidemiology—*What are the expected levels of risk and oral health in the covered population?*

If oral health (prevalence of disease) changes so do provider requirements.

Q/H: Evidence‐based services—*What services do we plan to provide for different risk/health groups?*

If services required to address disease change so do provider requirements.

N/Q: Productivity—*How do we plan to provide those services?*

If provider productivity (Q/N) changes, provider requirements change in the opposite direction.

## 6 APPRAISING THE ‘FIT FOR PURPOSE’ MODEL

It is odd that while several countries have adopted population needs–based approaches for distributing available funds for delivering health care between regional populations (ie, using needs as a basis of ‘slicing the healthcare cake’), the level of resources (services, workforce and funding) allocated to health care (or the ‘size of the cake’) has generally been determined by other factors unrelated to needs.^23^ Improving system performance, through adoption of services based on evidence of effectiveness among populations with needs, will depend crucially on ensuring that resources are available in accordance with the level of needs. The model presented above provides a transparent mechanism for planning the oral health workforce for both high‐income countries dealing with epidemiological transition from widespread caries among children, and low‐ and middle‐income countries moving towards universal health coverage for rapidly increasing populations that is expected to lead to rapid increases in service needs.^23^ Compared with existing health workforce planning models, the needs‐based model is aligned directly to the objective of healthcare systems, meeting the needs of populations and patients, and relates service and workforce levels to needs explicitly. In this way, the approach ensures that estimated workforce requirements reflect needs for care in the population, responds to changes in needs over time and incorporates funders’ planned responses to those needs, including prioritizing which needs will be addressed within expected resource constraints. The model is applied by building up a picture of needs within subgroups of the population to reflect the differing levels of oral health and needs for care between these groups (eg, young children, adolescents, working‐age adults and the elderly population). In this way, the model uses a ‘bottom‐up’ approach to present an overall view of the requirements of the system based on the requirements for these different population groups.

Under this needs‐based approach, increases in health workforce can only be justified where needs increase, the services planned to be provided for the same needs increase and/or provider productivity falls. Likewise, a contraction in the supply of the workforce can be planned in response to evidence of reductions in the prevalence of conditions, rationalization of services provided and improvements in productivity. However, this requires an iterative process of applying dynamic planning models that are frequently refreshed with the latest data and estimates of future values of the determinants of workforce requirements.

In addition to adopting needs as the basis of planning, the new approach integrates workforce planning and service planning, with the former being derived from the latter. Moreover, the model can be extended to include an additional cost component to relate workforce and service planning to healthcare expenditure planning, providing a clear and transparent basis for decisions about healthcare expenditure.

The model has some limitations including the data required for implementation. Although ‘perfect’ data might not currently be available for each element of the model, the approach can be used to start conversations about how we might reconsider the data to be collected. Planning for the future ideally uses information on the future. While we do not have a window into the future to know precisely what the values will be for the four elements in the model, we do have models and study designs (eg, cohort models) that help us provide more meaningful expectations of the future.^24^, ^25^, ^26^ These approaches represent a major advance on existing approaches such as ‘expert panels’, as they represent systematic ways of building on the accumulated lifetime experiences of population and provider groups using dynamic models.

A second limitation is that the model focusses on planning the ‘right’ capacity of a system to deliver the services required in a population based on system goals, population needs, best practice services and efficient models of service delivery but does not address directly the distribution of oral health services and the providers to deliver those services *within* a jurisdiction. Issues concerning the efficient *distribution* of the capacity to deliver services can be addressed by decentralizing capacity and workforce planning to smaller population units within a larger heterogeneous population. However, moving to smaller planning populations removes the benefits of economies of scale. Instead, system‐level workforce planning can be complemented with other policies (eg, provider payment methods, contracts for service provision for particular populations or settings, mobile services, teledentistry, artificial intelligence) aimed at directing or distributing workforce capacity, or developing new models of care within jurisdictions, to respond to differences in need, service opportunities and production constraints that occur between population settings.

If operationalizing the model for purpose of universal health coverage, it will be important to incorporate context‐specific input parameters which reflect the values of the respective population in terms of what constitutes access to good quality oral health care without financial hardship. To this end, conceptualizations of quality of oral health care and oral health investment case modelling have recently been proposed, both endorsing the utmost relevance of planning the oral health workforce according to people's health needs.^28^, ^29^, ^30^

## 7 CONCLUSIONS

Innovating planning for health care represents a disruptive translational technology aimed at bringing ‘knowledge to action’ and provides a direct response to sustainable development goals for health, particularly in terms of delivering safe, efficient, sustainable and equitable (oral) health care.

Only through such an integrated approach can we examine and plan different skill mixes and opportunities for workforce substitution as workforce requirements emerge from needs‐based service requirements and the models of delivery used to deliver those services. This contrasts with the silo‐based approaches of traditional workforce planning models which plan for doctors, dentists, nurses, etc, independently, even though these different workforces contribute together to the delivery of services. This incorporates (a) using a strategic approach to the use of the existing workforce and (b) planning the future workforce that develops policies for the number and types of providers to be trained for the future in the context of the available supply of healthcare providers and the expected changes in total workforce requirements.

Application of the model extends beyond planning healthcare services and resources through identifying the drivers of healthcare expenditure growth. This provides accountability to the differing claims about the causes of utilization and expenditure growth, as well as a tool for managing sustainability in healthcare systems. While technological change is often presented as a way of increasing system efficiency, its adoption often leads to rapid increases in utilization and expenditure. For example, telemedicine (the use of technologies to remotely diagnose, monitor and treat patients) and telehealth (the application of technologies to help patients manage their own illnesses through improved self‐care and access to education and support systems) are widely recognized as key strategies for improving health system efficiency. Yet, the adoption of these new technologies often leads to rapid and unplanned increases in utilization and expenditure because the potential efficiencies, in terms of reducing the requirements for other services as a result of the technologies, are not realized. The needs‐based model highlights the importance of a systems approach to managing what services are provided, and how they are provided, in the context of improving technology.

Given that training of oral healthcare professionals involves costly long‐term programmes, improving planning through the use of more appropriate data will generate significant benefits in terms of ensuring more accurate estimates of a system's health human resource requirements.

The four policy variables identified in section 5 provide an agenda for innovation in planning health workforces. Addressing these questions would generate the information required to implement needs‐based workforce planning. Such innovation in planning is required in order to move away from inherently inefficient models based predominantly on maintaining current population‐provider ratios, or uninformed reactions to perceived problems of access to care. If we do not take this opportunity, we risk failing to realize the potential gains from technological change, evidence‐based practice and improvements in understanding the social determinants of health.

## AUTHOR CONTRIBUTIONS

Stephen Birch led the conceptualization of the manuscript and the model development. He drafted the initial manuscript. All authors contributed to the conceptualization of the manuscript, identification of relevant literature, development of the model and provided comments on the initial and subsequent drafts of the manuscript.

## Data Availability

Data sharing not applicable – No new data generated, or the article describes entirely theoretical research.
